# Live birth following transfer of a euploid blastocyst derived from a 5PN zygote: a case report

**DOI:** 10.1007/s10815-026-03899-x

**Published:** 2026-05-16

**Authors:** Claudia Gomes, Mariana Nicolielo, Bruna Lourenço, Dayane Guerino Reis, Beatriz Aiello, Izadora Reis, Leticia Tamashiro, Andrea Belo, Hanna Park, José Roberto Alegretti, Dóris Spinosa Chéles, Aline Rodrigues Lorenzon

**Affiliations:** 1 Clinical Department, Huntington Medicina Reprodutiva - Eugin Group, São Paulo, Brazil; 2 Embryology Laboratory, Huntington Medicina Reprodutiva - Eugin Group, São Paulo, Brazil; 3 R&D Department, Huntington Medicina Reprodutiva - Eugin Group, São Paulo, Brazil

**Keywords:** 5PN zygote, Abnormal fertilization, PGT-A, Live birth

## Abstract

**Background:**

Abnormal pronuclear formation, including five pronuclei (5PN), is generally considered indicative of abnormal fertilization (e.g., polyspermy or failure of polar body extrusion) and leads to routine embryo discard in many IVF programs. However, emerging evidence suggests that a minority of embryos with atypical pronuclear patterns may develop into a blastocyst and present diploid constitution after genetic testing, challenging the assumption that abnormal pronuclear morphology invariably predicts non-viability.

**Case report:**

We report a rare case in which a 5PN zygote developed into a morphologically high-quality blastocyst was confirmed euploid–diploid by PGT-A, transferred in a subsequent frozen embryo transfer cycle, and resulted in the live birth of a healthy child.

**Conclusion:**

This case adds to the limited but growing evidence that embryos with atypical pronuclear presentation may retain reproductive potential when development on time-lapse appears coherent and validated genetic testing confirms diploidy and euploidy, supported by appropriate patient counseling and informed consent.

## Introduction

Normal human fertilization is typically identified by the presence of two pronuclei (2PN) and two polar bodies. Zygotes with atypical pronuclear (PN) counts, such as 0PN, 1PN, 3PN, or higher, are generally regarded as abnormally fertilized and therefore presumed to carry high rates of chromosomal imbalance, including triploidy or polyploidy [1]. For this reason, many laboratories discard embryos originating from ≥ 3PN zygotes, particularly when more typical embryos are available for transfer or cryopreservation [2].

However, current consensus highlights the limitations of morphology-based fertilization assessment. The revised Istanbul consensus emphasizes that static PN evaluation represents a temporal snapshot and may not fully capture the dynamic nature of pronuclear formation and syngamy, potentially leading to misclassification of fertilization status [3].

Consistent with this, emerging evidence indicates that abnormal PN morphology does not invariably correspond to irreversible developmental failure. Studies on monopronuclear (1PN) embryos, including recent data by Bartolacci et al. [4], have demonstrated that a subset of these embryos can develop to blastocyst stage and present diploid chromosomal constitution, challenging the assumption that atypical PN patterns necessarily reflect abnormal ploidy or lack of developmental competence [4].

A small proportion of embryos derived from abnormally fertilized oocytes may progress to blastocyst and display diploid constitution by modern genetic assessment, raising the possibility of careful, selected use under specific circumstances [1–8]. Capalbo et al. [1] specifically emphasized that contemporary PGT-A platforms incorporating ploidy assessment can support the identification of rare viable embryos among those that would otherwise be routinely discarded.

Here, we report a rare case of a 5PN zygote that developed into a high-quality blastocyst, was confirmed euploid–diploid through PGT-A, transferred, and resulted in the birth of a healthy child. To our knowledge, live birth following transfer of a blastocyst derived from a 5PN zygote has not been previously reported, highlighting the rarity of this observation. This case adds to the growing body of evidence that embryos with atypical pronuclear presentations may, in select circumstances, exhibit normal developmental potential and reproductive competence.

## Case report

In November 2023, a 35-year-old woman and 41-year-old man were admitted to our clinic due to infertility for the last 3 years. She had a laparoscopy for deep endometriosis resection and fimbrioplasty in 2022 and he had a varicocelectomy in 2021. After the laparoscopy, they were submitted to an ovulation induction with letrozol 5 mg/day for 4 months with no success. They were admitted into our clinic with an indication to perform an in vitro fertilization (IVF) cycle. At their first consultation, her anti-Müllerian hormone (AMH) level was 0.49 ng/mL, her antral follicular count was 10, and her thyroid-stimulating hormone (1.52 µUI/mL) and prolactin levels (12.03 ng/mL) were normal. She had low free testosterone levels (0.07 pg/mL). Her transvaginal ultrasound, Pap smear, and mammography results were normal. Her body mass index was 22.3 kg/m^2^. Thrombophilia tests and pelvic magnetic resonance imaging (MRI) were normal. Her partner’s medical history revealed hepatic steatosis with alanine transaminase and aspartate transaminase (ALT and AST) alterations for which he was under clinical treatment. His body mass index was 31.3 kg/m^2^. Semen analysis and semen DNA fragmentation were normal. The couple’s karyotypes were normal.

Antioxidant supplements to minimize age-related oxidative stress were prescribed for both, including 800 mg/day of coenzyme Q10 and 12.5 mg/day of testosterone gel for the woman, for 60 days before egg retrieval to increase follicular FSH responsiveness. In March 2024, ovulation induction of 10 antral follicles was started with hMG 300 UI at day 2 of the menstrual cycle. Ganirelix acetate 0.25 mg a day was prescribed for ovarian stimulation and inhibition of the LH surge when at least one follicle had 13–14 mm. On day 10 of ovarian stimulation, oocyte maturation was triggered with hCG 250 mcg, 35 h before the egg retrieval.

**Fig. 1:**
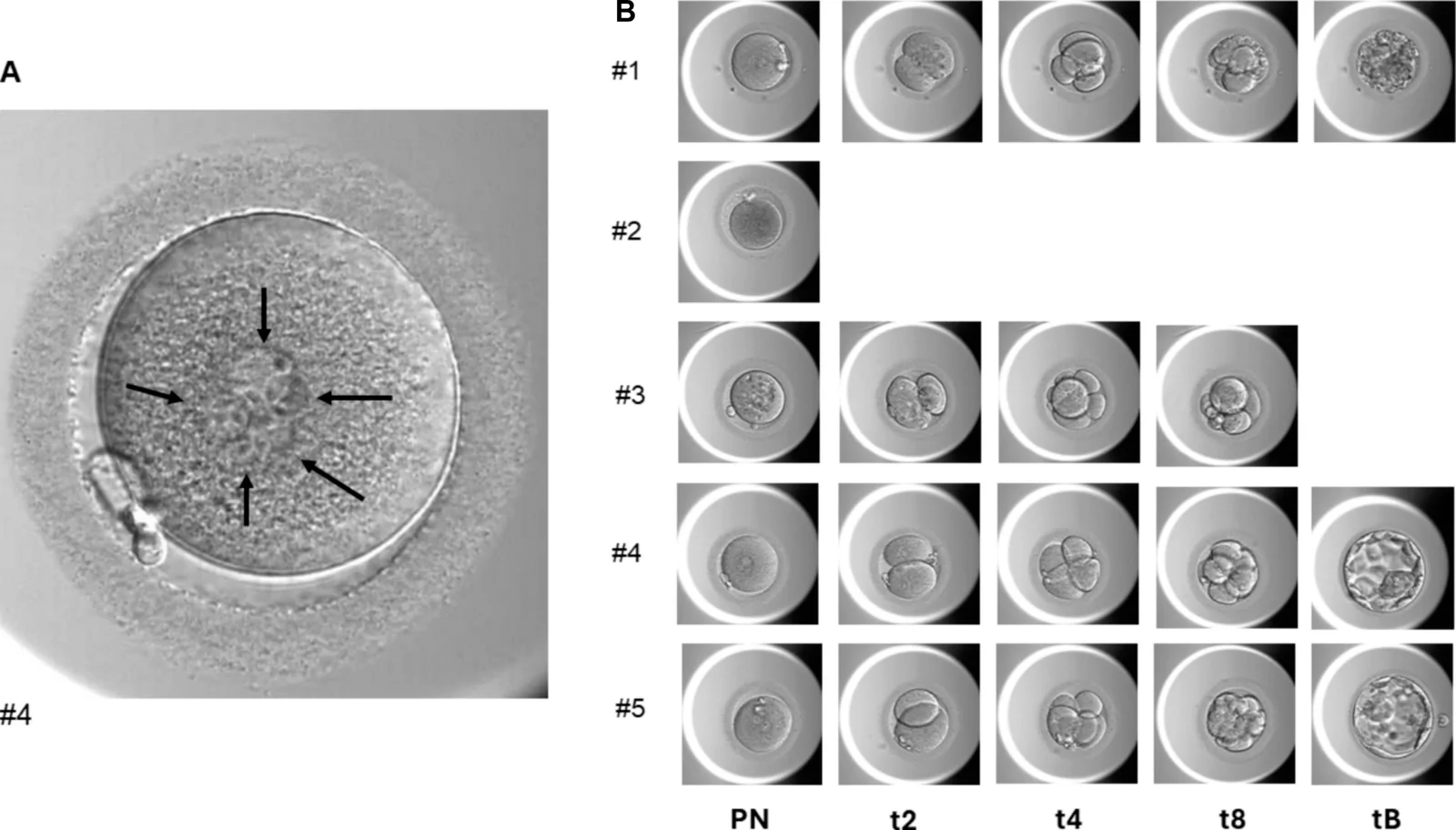
Pronuclear assessment and time-lapse development of embryos from the reported cycle.** A** Representative image of the five-pronuclear (5PN) zygote; arrows indicate the five pronuclei. **B** Time-lapse images of embryos #1–#5 at key developmental time points: pronuclear stage (PN), 2-cell (t2), 4-cell (t4), 8-cell (t8), and blastocyst (tB) (times annotated relative to ICSI, t0). Embryo #1 arrested at the early blastocyst stage (Gardner stage 2), Embryo #2 arrested after the PN stage, #3 arrested at cleavage stage, whereas embryos #4–#5 progressed to blastocyst

The male semen parameters before preparation were sperm count of 23 × 10^6^ million/mL, a total sperm progressive count of 8 × 10^6^ million/mL (34.8%), and a Kruger’s morphology of < 1%. Viscosity was increased, and the sample presented numerous gelatinous bodies. After sperm preparation with discontinuous density gradient, the sperm concentration and the progressive count were 5 × 10^6^ and 3 × 10^6^ million/mL respectively, and Kruger’s morphology was < 1%. Six eggs were retrieved and denuded after 3 h to assess maturation. Three were metaphase II (MII), 2 at metaphase I (MI) that matured in vitro to MII and 1 at germinative vesicle (GV) stage. In case of metaphase I (MI) stage, oocytes were placed in continuous single culture media (CSC-NX Complete®, Irvine Scientific) for additional 6 h to reach MII stage. Mature oocytes were fertilized by IMSI (intracytoplasmic morphologically selected sperm injection) due to poor semen morphology. At day 1, four embryos were formed—#1 3PN +, #3 2PN, #4 5PN (Fig. 1A), #5 2PN—resulting in two blastocysts (#4 5BA and #5 4BB, morphological grading according to Gardner and Schoolcraft scale) at day 5 of embryo culture in a time-lapse incubator (Embryoscope Plus™, Vitrolife) and continuous single culture media (CSC-NX Complete®). Both blastocysts (#4 and #5) originated from oocytes that were initially at the MI stage and subsequently matured in vitro (rescue IVM), whereas embryos derived from in vivo matured MII oocytes arrested prior to the blastocyst stage (Fig. 1A and B). Notably, the 5PN zygote that resulted in a euploid blastocyst and live birth originated from an oocyte that underwent rescue IVM.

Both embryos were subjected to trophectoderm (TE) biopsy followed by preimplantation genetic testing for aneuploidy (PGT-A plus, Igenomix São Paulo, Brazil). After trophectoderm biopsy, blastocysts were vitrified using the INGAMED vitrification kit according to the manufacturer’s protocol. Embryo #4 was reported as euploid (46XY, morphology classification 5BA) and evaluated through artificial intelligence (AI) tools, KIDScore™: 6.5 and Morphological Artificial Intelligence Assistance (MAIA) score: 7.28 [9]. The other blastocyst was aneuploid (embryo #5, 45XX-21, 4BB, 3.4 and 0.97, respectively).

As the only euploid blastocyst available originated from a 5PN zygote, the couple was thoroughly counseled regarding the atypical fertilization pattern, the associated biological uncertainties, and the inherent limitations of PGT-A, including the possibility of undetected chromosomal or genetic abnormalities. After detailed discussion of the potential residual risks, written informed consent was obtained prior to embryo transfer.

On March 2024, uterine preparation was started on day 2 of the menstrual cycle with 2 mg of valerate estradiol twice a day for 3 days and then 4 mg of valerate estradiol twice a day for more than 3 days, but the endometrial lining was not adequate (hyperechogenic aspect), and progesterone levels increased prematurely; therefore, embryo transfer was cancelled.

On May 2024, 4 mg of valerate estradiol every 12 h was prescribed on day 3 of the menstrual cycle. After 13 days of hormonal therapy, the endometrial lining was 8.0 mm long, trilaminar and no dominant follicle was detected by ultrasound, estradiol levels were 215 pg/mL, and progesterone levels were 0.5 ng/mL. Vaginal progesterone (1200 mg/day) and dydrogesterone 30 mg/day were prescribed. Embryo #4 was warmed on the day of transfer using the corresponding INGAMED warming kit, approximately 3 h prior to embryo transfer. Full post-warming survival and re-expansion were observed prior to transfer. Embryo transfer was performed on day 6 of progesterone administration. Estradiol level was 258 pg/mL, and her progesterone level was 15.3 ng/mL on the day of embryo transfer. There were no technical difficulties with the embryo transfer procedure.

The first quantitative beta-hCG was measured 10 days after embryo transfer, and the result was 277.9 mUI/mL. Luteal support was continued until the eighth week of gestation, when the medication dosage progressively decreased and completely stopped at 12 weeks of gestation. First- and second-trimester morphological ultrasound data, including uterine artery Doppler data, were normal for all risk markers. The patient had an ongoing pregnancy without any complications, and acetylsalicylic acid (100 mg/day) was used for preeclampsia prevention. Blood pressure started to increase during 38 weeks of gestation and a nonlabored C-section was recommended by her obstetrician. A healthy baby boy, with a birth weight of 3168 g and length of 51 cm, was born in late February 2025 at 38 weeks and 5 days of pregnancy. The APGAR scores at 1 and 5 min were 9 and 10, respectively. The patient and baby were discharged from the hospital after 48 h.

Informed consent was obtained from the subjects of this case report.

## Discussion

This case documents a live birth following transfer of a blastocyst derived from a 5PN zygote that was reported as euploid–diploid by PGT-A. In most IVF laboratories, embryos originating from ≥ 3PN zygotes are routinely discarded because abnormal PN formation is strongly associated with chromosomal abnormalities, including triploidy/polyploidy, and presumed lack of reproductive potential [2]. However, this case adds to the limited evidence indicating that a small subset of embryos with atypical PN patterns may progress to blastocyst and present diploid constitution when assessed with modern genetic technologies [1, 2, 10].

Healthy live births from embryos originating from abnormal fertilization patterns remain uncommon but have been documented for some 3PN and 4PN-derived blastocysts, particularly when ploidy assessment confirms diploidy [5–8, 11]. A recent study highlighted that while most embryos derived from abnormal zygotes show chromosomal abnormalities, a small proportion may present normal ploidy, suggesting that abnormal pronuclear morphology does not invariably indicate irreversible developmental failure [10].

Capalbo et al. [1] proposed that enhanced PGT-A platforms capable of reliable ploidy assessment can help identify rare viable embryos among those labeled “abnormally fertilized,” potentially enabling clinical use under carefully controlled conditions. Likewise, a scoping review summarized that although most abnormally fertilized embryos show chromosomal abnormalities, diploid outcomes are occasionally reported, particularly when embryos develop to blastocyst and undergo genetic evaluation [2]. Our report extends this evidence to a rarer PN phenotype (5PN), emphasizing that PN count alone may not be sufficient to determine embryo fate in all cases.

The biological explanation for how a 5PN zygote could yield a diploid blastocyst remains uncertain. Proposed mechanisms in the literature include atypical PN configuration, asynchronous PN formation, or post-zygotic events leading to diploid rescue; however, these remain hypothetical and cannot be confirmed from this single case [5]. Importantly, this report should not be interpreted as suggesting that 5PN embryos are frequently viable. Rather, it supports a cautious “conditional continuation” approach: if an embryo derived from atypical PN morphology shows development consistent with expected morphokinetic parameters and reaches the blastocyst stage and validated genetic testing confirms diploidy and euploidy, the embryo may be considered for clinical use following thorough counseling and explicit consent. This approach may be particularly relevant in patients with limited embryo availability, such as poor responders, where strict discard policies based solely on pronuclear morphology could further reduce already constrained reproductive opportunities.

From a practical standpoint, the decision in this case was supported by a convergence of risk-mitigation measures: (i) high-quality blastocyst development, (ii) time-lapse monitoring documenting coherent morphokinetics, and (iii) euploid–diploid PGT-A results. AI-based embryo scores may provide additional supportive information, but genetic confirmation and development to blastocyst remain the primary anchors for safety when considering atypical PN embryos. [1, 2]

This case has limitations. It represents a single observation, and we cannot determine the true frequency of euploid outcomes among 5PN embryos or identify predictive morphokinetic patterns from one embryo. Additionally, while neonatal outcome was normal, postnatal genomic testing was not performed. Nonetheless, the report highlights an important question for laboratory policy: whether universal discard of embryos solely based on PN morphology may occasionally exclude embryos with reproductive potential.

Future work should focus on quantifying the frequency of diploid/euploid outcomes among embryos derived from atypical PN zygotes using standardized methods, defining developmental/morphokinetic features associated with diploidy, and establishing consensus criteria for any conditional rescue approach that ensures patient safety, ethical oversight, and clear counseling [1, 2].

## Data Availability

Not applicable. According to local institutional policy, this case report did not require Ethics Committee review. Written informed consent for publication was obtained.
